# Comparison of External Circular Fixation and Plate and Screw Fixation Methods in Tibial Pilon Fractures

**DOI:** 10.1055/s-0044-1790213

**Published:** 2024-12-07

**Authors:** Marco Antonio Schueda, Leonardo Maranhão Gubert, Yan Celuppi Dal Vesco, Fernanda Fossa Dal Piva, Felipe Guglielmi Niada, Ryad Fayez Mehanna

**Affiliations:** 1 Serviço de Ortopedia e Traumatologia, Hospital do Coração Balneário Camboriú, Balneário Camboriú, SC, Brasil; 2 Serviço de Ortopedia e Traumatologia, Hospital e Maternidade Marieta Konder Bornhausen, Itajaí, SC, Brasil; 3 Serviço de Ortopedia, Hospital Unimed Litoral, Balneário Camboriú, SC, Brasil

**Keywords:** external fixators, Ilizarov technique, tibial fractures

## Abstract

**Objective** To compare the fixation methods for tibial pilon fractures among patients treated in a hospital.

**Methods** We analyzed the medical records of 28 patients who underwent a surgical procedure for tibial pilon fracture, among whom 15 subjects received a circular external fixator, and 13 underwent internal fixation using a plate and screws. We assessed age, sex, aggravating factors, trauma energy (high or low), presence of soft tissue injuries, associated fractures, and clinical outcomes.

**Results** Most patients were male, aging between 40 and 60 years. The most common trauma mechanism was car accident, and the associated injury was a fracture of the distal third of the fibula. The fracture patterns in patients treated with a circular external fixator were AO 43C3 and 43C2. As for the prevalent fracture patterns in the internal fixation group, we identified AO 43C1, 43C2, and 43C3.

**Conclusion** An individualized therapeutic choice is critical for a better functional outcome. Additionally, it is essential to highlight that the profile of fractures and patients in the circular external fixator and internal fixation groups tends to be quite heterogeneous, because the treatment of fractures with the worst classification and most frequently associated with soft tissue injuries often uses circular external fixation; meanwhile, those with less severe fractures and a lower incidence of soft tissue injuries are usually managed with open reduction and internal fixation. We noted that the clinical and radiographic outcomes tended to be similar between both groups despite the particularities of each method.

## Introduction

The term tibial pilon was introduced in 1911 by the French radiologist Destot, who described fractures involving the weight-bearing joint surface of the distal third of the tibia but not necessarily affecting it. 1 Radiographic evaluation requires three views: anteroposterior (AP), lateral (L), and AP with 20° of internal rotation (mortise). In cases of doubt, one may request an oblique or stress radiograph. Additionally, one may ask for a radiograph of the contralateral ankle for comparison. In cases of joint involvement, especially in fractures caused by high-energy trauma, computed tomography (CT) helps to evaluate the fracture pattern and joint comminution degree, as well as preoperative planning. 2

Tibial pilon fractures predominantly affect young men between 30 and 40-years-old, and high-energy trauma accounts for 10 to 30% of open fractures. 3 Currently these fractures are common because of high-energy traumas, such as traffic accidents or falls from height. Other fractures, including in the talus, calcaneus, tibial plateau, pelvis, acetabulum, and spine, may accompany tibial pilon fractures. 4

The trauma mechanism is longitudinal compression of the talus on the distal surface of the tibia, potentially associated with rotational forces. The fracture pattern results from the direction and speed of the damaging energy, along with the foot's positioning during load application. 5

Furthermore, it is important to assess soft tissue involvement and consider whether the fracture is open or closed. We know many factors deserve consideration when choosing the definitive treatment 6 since the soft tissue involvement may suffer exacerbation by major surgical procedures involving large incisions, bone deperiostization, and separation of its soft components, which can lead to necrosis of the overlying tissue and increase the risk of pseudarthrosis, infection, or both. 7

## Materials and Methods

Our institution approved this study under CAAE number 71275623.0.0000.0120.

This cross-sectional and observational study analyzed the medical records of patients hospitalized with a tibial pilon fracture who underwent surgical treatment from January 1st, 2021, to January 1st, 2023. Using the medical records, we assessed age, sex, aggravating factors, trauma energy, presence of soft tissue injury, and occurrence of associated fractures. We analyzed imaging tests (AP, L, and oblique radiographs of the ankle and preoperative CTs), transoperative, immediate postoperative, and postoperative follow-up radiographies in ambulatory. For the functional analysis, the American Association for Foot and Ankle Surgery's (AFOS) functional scores were used.

Polytrauma patients underwent damage control treatment with mono or biplanar fixation and a review for internal or external circular fixation within 7 days of their emergency treatment.

## Results

We analyzed the medical records from 30 patients operated on for tibial pilon fracture, including 15 treated with a circular external fixator and 15 treated with internal fixation using a plate and screws. We lost 2 patients from the internal fixation group at follow-up and excluded them from the analyses. Thus, this subgroup had 13 patients, and the total research sample comprised 28 subjects.

**Fig. 1:**
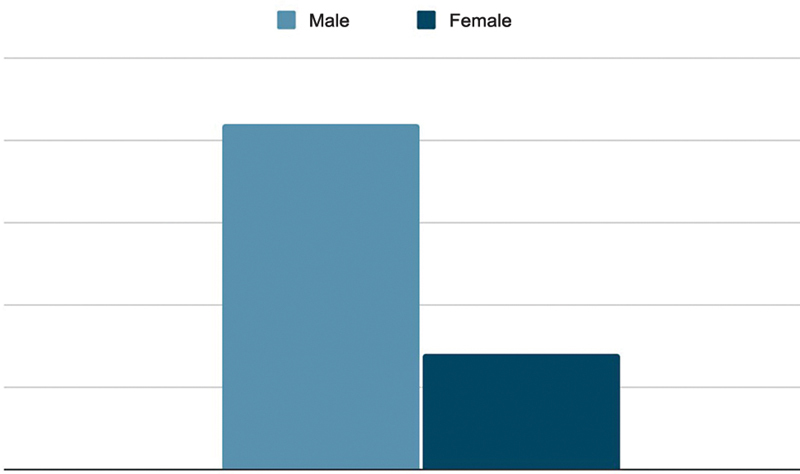
Sex of the participants.

The patients included 21 males and 7 females ( Fig. 1 ). The external fixation subgroup treated using the method proposed by Ilizarov had 15 patients, including 10 males and 5 females. The internal fixation subgroup treated using a nonlocking trefoil plate and screws had 13 patients, with 11 males and 2 females.

**Fig. 2:**
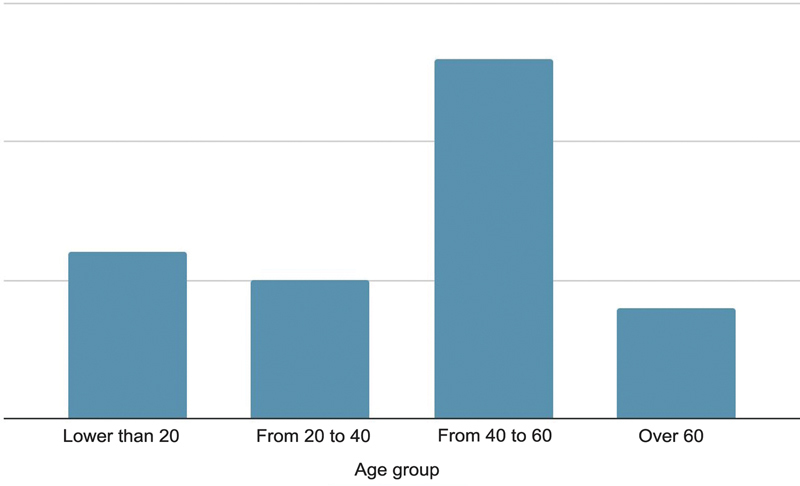
Age of the patients.

We observed a high prevalence of patients in the age group from 40 to 60-years-old, with an average of 44 ( Fig. 2 ). The average age was 43 in males and 46 in females.

Regarding the mechanism of trauma in the circular external fixator group, we observed a higher tendency for high-energy trauma (11 patients). Car accidents accounted for 7 of the 11 cases, while the remaining 4 resulted from falls from height. From that last group, all 4 patients suffered lower-energy trauma resulting from falls from their height. Among patients from the circular external fixator group, there were 60 open and 40% closed fractures.

**Fig. 3:**
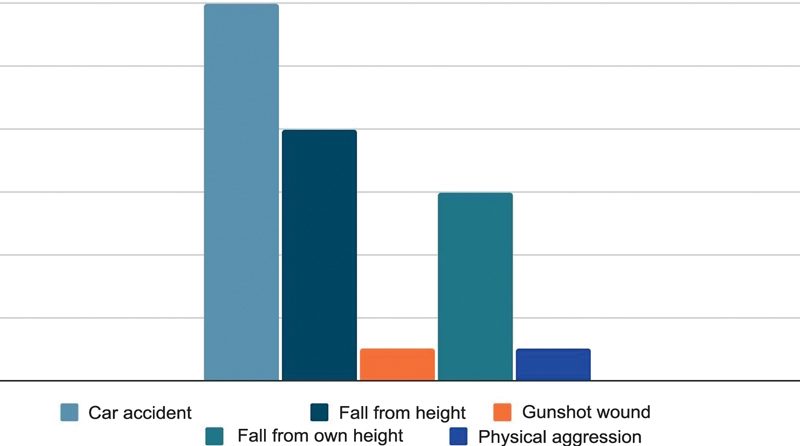
Mechanism of trauma.

Regarding the trauma mechanism in the internal fixation group, a high tendency towards high-energy trauma was noted (10 patients). Car accidents accounted for 5 of the 10 cases, while another 4 cases resulted from falls from height, and 1 involved a gunshot wound. Among the 3 patients suffering low-energy trauma, 2 had fractures from physical aggression, and 1 case was due to a fall from their level ( Fig. 3 ). Therefore, the internal fixation group had approximately 77% closed and 23% open fractures.

According to the AO group classification for fracture patterns, we noted that 60% of patients treated with a circular external fixator had 43C3 fractures with frank joint comminution, and another 40% had 43C2. No subject treated with the circular external fixation method had 43C1 fractures.

Using the same classification, we observed that patients treated with internal fixation included 5 subjects with 43C1 fractures, 5 with 43C2 fractures, and 3 with 43C3 fractures.

In both groups, the associated injury in 100% of cases was a fracture of the distal third of the fibula. Other injuries in the circular external fixation group included tibial plateau (4 cases), calcaneus (2 cases), tibial diaphysis, contralateral ankle, and hand bone fractures (1 case each). A clavicle fracture was the single other injury observed in the internal fixation group.

**Fig. 4:**
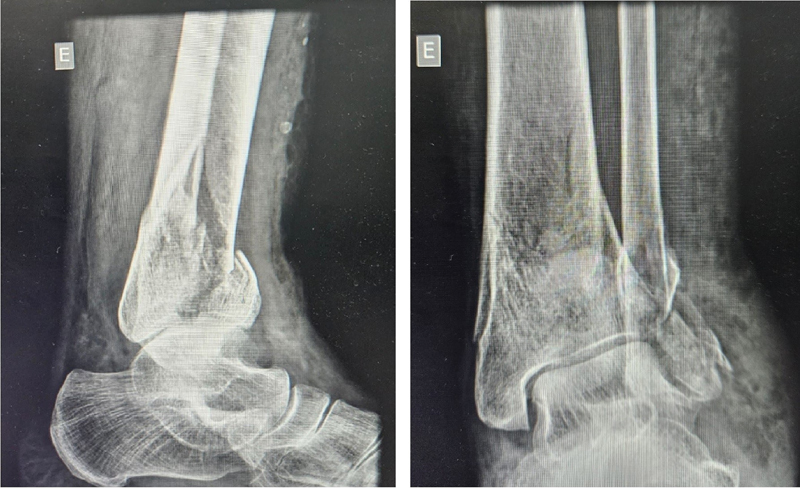
Tibial pilon fracture (AO 43C2) before internal fixation with plate and screw.

Regarding the outcomes of the circular external fixator group, 5 patients presented complete fracture consolidation, 1 had pseudarthrosis, and 2 malunions. Figs. 4 5 6 7 show examples of the circular external (I) and internal fixation (II) groups.

**Fig. 5:**
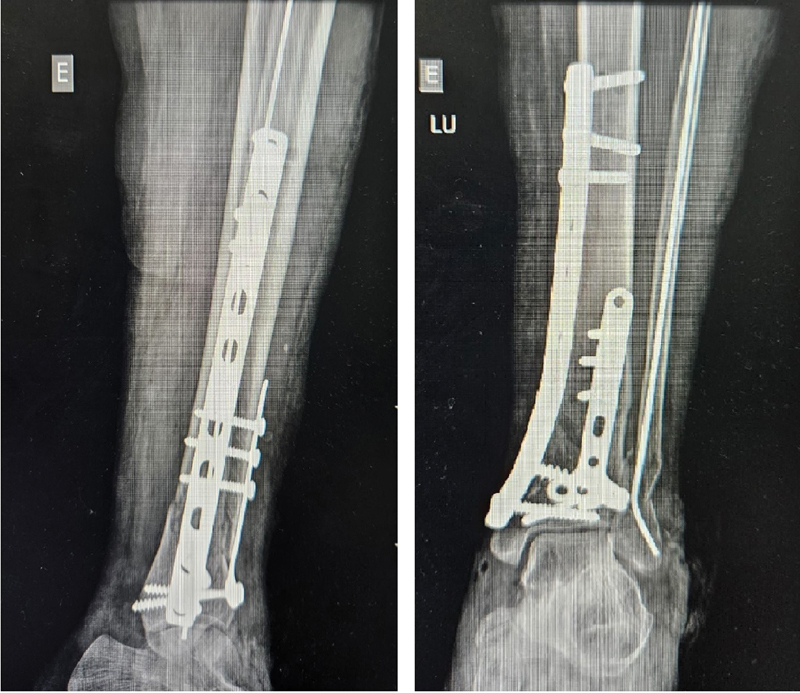
Tibial pilon fracture (AO 43C2) after internal fixation with plate and screw.

**Fig. 6:**
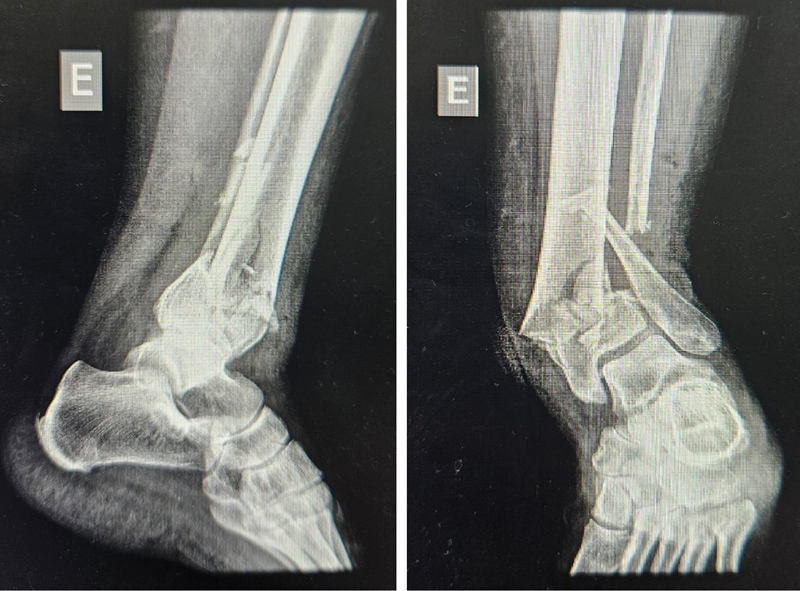
Tibial pilon fracture (AO 43C2) before circular external fixation.

**Fig. 7:**
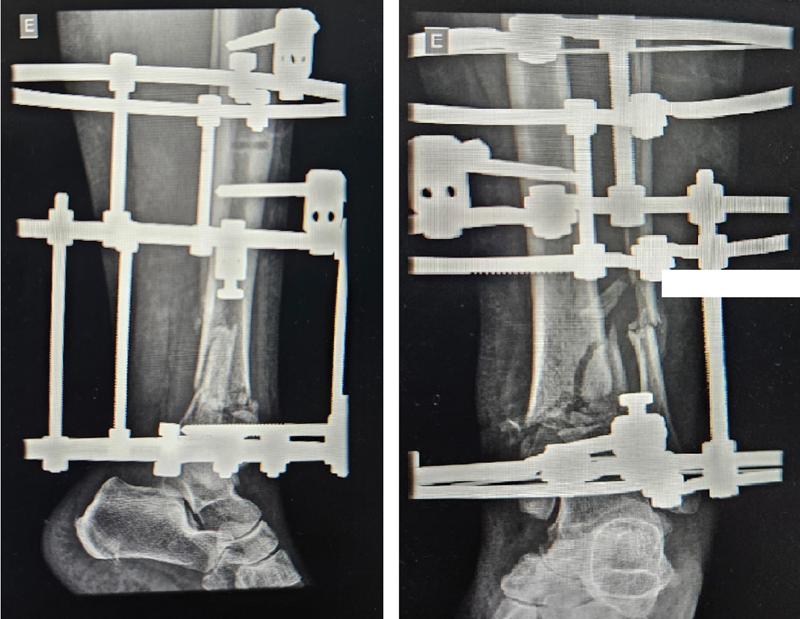
Tibial pilon fracture (AO 43C2) after circular external fixation.

**Fig. 8:**
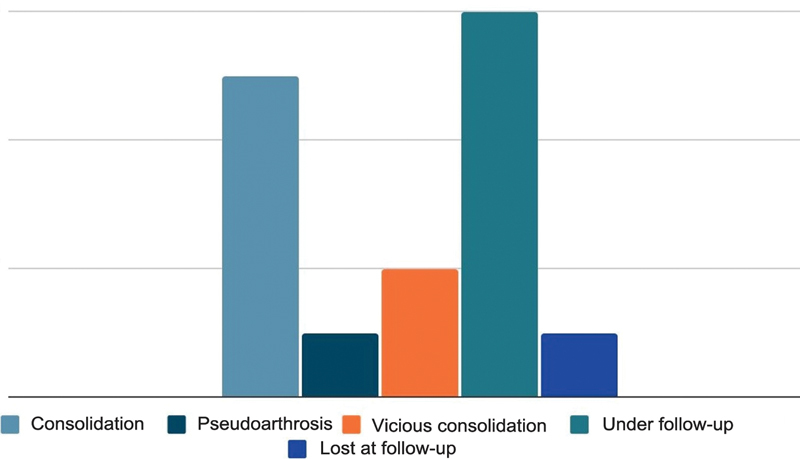
Circular external fixation outcome.

A total of 6 patients still remain in follow-up, but their progress is satisfactory, with radiographic consolidation signs. A single patient was lost at follow-up ( Fig. 8 ).

**Fig. 9:**
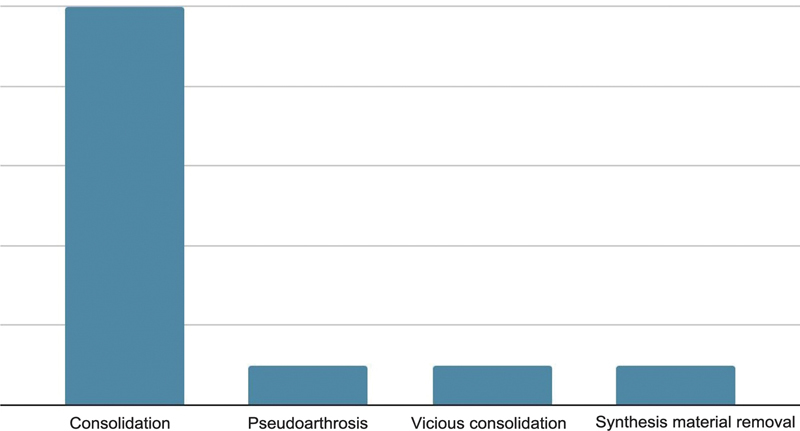
Internal fixation (plate and screw) outcome.

As for the outcomes of the open reduction and internal fixation group, 10 patients presented complete fracture consolidation, 1 had pseudarthrosis, 1 presented with malunion. Finally, one of the cases had a frank infection in the topography requiring synthetic material removal and multiple surgical interventions ( Fig. 9 ).

**Fig. 10:**
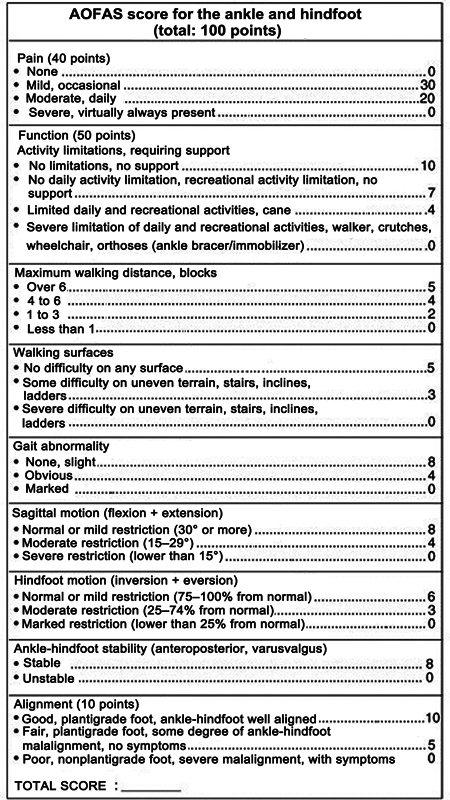
American Orthopedic Foot & Ankle Society's (AOFAS) score for the ankle and hindfoot (total: 100 points).

To facilitate the functional assessment of patients according to the American Orthopedic Foot & Ankle Society (AOFAS) score for the ankle and hindfoot ( Fig. 10 ), we subdivided the patients as follows:

- Group 1: 0 to 20 points
- Group 2: 20 to 40 points
- Group 3: 40 to 60 points
- Group 4: 60 to 80 points
- Group 5: 80 to 100 points

As such, we noted that the distribution of the groups was as follows: group 1–2 patients; group 2–6 patients; group 3–7 patients; group 4–8 patients (this group presented the highest prevalence); and group 5–5 patients.

The group treated with the Ilizarov external fixator contained 15 patients, distributed as follows: group 1–1 patient; group 2–3 patients; group 3–4 patients; group 4–4 patients; and group 5–3 patients.

In contrast, the group treated with internal fixation (plate and screws) comprised 13 patients with the following distribution: group 1–1 patient; group 2–3 patients; group 3–3 patients; group 4–4 patients; and group 5–2 patients.

## Discussion

Our study revealed a prevalence of males, which is consistent with the literature. However, the prevalent age group was older than the one reported by Marsh and Saltzaman. 5 Pimenta et al. 8 observed an average age of 42, similar to our study.

In the circular external fixator group, the rate of open fractures was above the percentiles found in the literature. 9 In contrast, the internal fixation group had a rate of soft tissue injuries with fracture exposure well below the literature, in which open fracture rates are at around 50%.

In our service, the main mechanism of trauma was car accidents, followed by falls from a height, which is consistent with the literature. Furthermore, it was observed that the highest rate of fracture exposure among the 28 patients occurred in the group classified as AO 43C2, representing 60% of subjects, compared to 38% in the AO 43C3 group. These rates differ from the literature, which reports a higher percentile of open fractures in the group with the most complex fractures, classified as AO 43C3. 2

We detected no statistically significant difference when evaluating the presence of a fibula fracture and correlating it with high- and low-energy traumas. This lack of difference occurred because, in our study, all patients presented an associated distal fibula fracture, even those suffering low-energy traumas. Furthermore, the literature reports that ipsilateral talus and calcaneal fractures accompanying tibial pilon fractures are extremely rare. However, we had 2 patients with concomitant calcaneal fractures, which may negatively influence long-term outcomes. 9 10

Regarding radiographic outcomes, it appears that studies evaluated them differently. Here, we concluded that among patients receiving a circular external fixator and with a definitive radiographic outcome, 62.5% presented fracture consolidation, 25 had malunion, and 12.5 presented nonunion. Among those undergoing internal fixation, 84% presented union, 8% had malunion, and 8% presented pseudarthrosis. The full assessment of the remaining subjects was not feasible, leading to their exclusion.

As for this series' general median, considering all 28 patients, the average AFAOS score was 55.7 points (circular external fixator group: 56.6; internal fixation: 54.6). This score is lower than the average of 65 points reported by Moura Junior et al. 2

## Conclusion

The present study retrospectively assessed 28 cases of tibial pilon fracture, including 15 patients undergoing surgical treatment with a circular external fixator and 13 with internal plate fixation (simple, cloverleaf type, with no locking mechanism). There was a wide range of variants directly influencing the characteristics of each fracture and its clinical-radiographic outcome.

Although there are many therapeutic alternatives for tibial pilon fractures, the internal fixation methods using unlocked plates and screws, as well as the circular external fixation method, remain the most available, mainly in hospitals within the public health system. Therefore, discussing these methods is critical and inevitable.

It is essential to highlight that the profile of fractures and patients in the circular external fixator and internal fixation groups tended to be quite heterogeneous. This occurs because the treatment of fractures with worst AO classifications and more frequent association with soft tissue injuries (resulting from open fractures or presenting significant edema or blistering) often employs circular external fixation. Meanwhile, management of less-severe fractures, with a lower incidence of soft tissue injuries, usually relies on open reduction and internal fixation. Clinical and radiographic outcomes tended to be similar between both groups, despite the particularities of each method.

We conclude that it is critical to perform an attentive, individualized, and detailed assessment. This will enable a more assertive therapeutic choice, leading to better functional outcomes for the particularities of each case and lower rates of socioeconomic impact for those with a tibial pilon fracture.
