# Supplementary material for: Comparison of External Circular Fixation and Plate and Screw Fixation Methods in Tibial Pilon Fractures
Source: Rev Bras Ortop (Sao Paulo). 2024 Dec 7;59(5):e663–71. doi: 10.1055/s-0044-1790213 (PMC11624940; doi:10.1055/s-0044-1790213)
Supplement: Related texts

# Research article: Comparação entre métodos de fixação externa circular e de fixação com placa e parafuso nas fraturas do pilão tibial

**Keywords:** fixadores externos, fraturas da tíbia, técnica de Ilizarov

## Abstract

**Objetivo** Comparar os métodos de fixação de fraturas do pilão tibial entre pacientes atendidos em um hospital.

**Métodos** Foram analisados os prontuários de 28 pacientes que realizaram procedimento cirúrgico para fratura do pilão tibial, sendo 15 deles tratados com fixador externo circular e 13 com fixação interna por placa e parafusos. Foram analisados idade, sexo, fatores agravantes, energia do trauma (alta ou baixa), presença de lesão de tecidos moles, ocorrência de fraturas associadas e desfechos clínicos.

**Resultados** O sexo mais acometido foi o masculino, na faixa etária entre 40 e 60 anos. O mecanismo de trauma em sua maioria foi acidentes automobilísticos e a lesão associada em 100% dos casos foi a fratura do terço distal da fíbula. O padrão de fratura observado nos pacientes tratados com fixador externo circular foram AO 43C3 e 43C2. Já os padrões de fratura prevalentes observado no grupo de fixação interna foram AO 43C1, 43C2 e 43C3.

**Conclusão** A escolha terapêutica individualizada é de extrema importância para um melhor desfecho funcional. Também é imprescindível ressaltar que o perfil da fratura e dos pacientes do grupo fixador externo circular e de fixação interna por placa e parafusos tende a ser bastante heterogêneo, pois as fraturas de pior classificação e mais frequentemente associadas a lesões de partes moles costumam ser manejadas com fixação externa circular, enquanto aqueles com fraturas de menor gravidade e menor incidência de lesão de partes moles tendem a ser manejados por redução aberta e fixação interna. Observa-se que os desfechos clínicos e radiográficos tendem a ser similares entre ambos os grupos apesar das particularidades de cada método.

## Introdução

O termo pilão tibial foi introduzido em 1911 pelo médico radiologista francês Destot, que descreveu as fraturas envolvendo a superfície articular de sustentação do peso de terço distal da tíbia, embora, não necessariamente a acometam. 1 Para seu diagnóstico deve-se realizar avaliação radiográfica em três incidências: anteroposterior (AP), perfil (P) e AP com 20° de rotação interna (mortise). Em casos de dúvida, pode-se solicitar radiografia em oblíquo ou com estresse. Além disso, pode-se pedir uma radiografia adicional do tornozelo contralateral para comparação. Na presença de acometimento articular, principalmente nas fraturas por trauma de alta energia, a tomografia computadorizada (TC) é útil para avaliar o padrão de fratura e o grau de cominuição articular, bem como planejamento pré-operatório. 2

Sabe-se que as fraturas do pilão tibial acometem predominantemente homens jovens, entre 30 e 40 anos de idade, e trauma de alta energia compreende uma taxa de 10 a 30% de fraturas expostas. 3 Atualmente, essas fraturas são comuns devido a traumas de alta energia, como acidentes de trânsito ou queda de alturas. Dessa forma, podem estar associadas a fraturas do tálus, calcâneo, planalto tibial, pelve, acetábulo e coluna vertebral. 4

O mecanismo do trauma é a compressão longitudinal do tálus na superfície distal da tíbia, podendo estar associada as forças de rotação. O padrão de fratura resultante dependerá do sentido e da velocidade de aplicação da energia lesiva, assim como da posição do pé no momento da carga aplicada. 5

Além disso, é necessário considerar se a fratura é aberta ou fechada e avaliar o envolvimento dos tecidos moles. Hoje, já se tem conhecimento de que muitos fatores devem ser levados em consideração na escolha do tratamento definitivo, 6 visto que o acometimento das partes moles pode ser exacerbado por grandes procedimentos cirúrgicos envolvendo grandes incisões, desperiostização do osso e separação de componentes moles dele, o que pode levar à necrose do tecido sobrejacente e aumentar o risco de pseudoartrose e/ou infecção. 7

## Materiais e Métodos

O trabalho foi aprovado pela nossa instituição sob o número CAAE: 71275623.0.0000.0120.

Estudo transversal do tipo observacional, realizado perante a análise de prontuários dos pacientes, admitidos em Hospital, com fratura de pilão tibial, que realizaram tratamento cirúrgico no período de 1° de janeiro, 2021, até 1° de janeiro, 2023. A partir dos dados médicos, foram analisados em prontuário idade, sexo, fatores agravantes, energia do trauma, presença de lesão em tecidos moles e ocorrência de fraturas associadas. Foi realizada análise de exames de imagem (radiografia em AP, P e obliquas do tornozelo, e TC pré-operatórias) e radiografias trans-, pós-operatórias de controle e no acompanhamento pós-operatório em retornos ambulatoriais. Para avaliação funcional utilizou-se o escore funcional da Associação Americana de Cirurgia do Pé e Tornozelo (AFOS).

Referente ao tratamento na urgência os pacientes politrauma foram submetidos a tratamento para controle de danos com fixação mono ou biplanar sendo revisados para fixação interna ou circular externa em até 7 dias no seu tratamento emergencial.

## Resultados

Foram analisados os prontuários de 30 pacientes operados para fratura de pilão tibial, sendo 15 deles tratados com fixador externo circular e 15 tratados com fixação interna por placa e parafusos. Do grupo de fixação interna por placa e parafusos, 2 pacientes perderam o seguimento e não foram incluídos nas análises. Dessa forma, o subgrupo em questão contém 13 pacientes e o grupo total da pesquisa em 28 participantes.

Dentre os pacientes analisados, observou-se que 21 pacientes do sexo masculino e 7 do feminino ( Fig. 1 ). O subgrupo submetido a fixação pelo método proposto por Ilizarov foi composto por 15 pacientes, dos quais 10 eram do sexo masculino e 5 do feminino. O subgrupo submetido a fixação interna utilizando placa trevo não-bloqueada e parafusos foi composto por 13 pacientes, dos quais 11 eram do sexo masculino e 2 do feminino.

**Fig. 1:** Sexo dos participantes.

Observou-se uma alta prevalência da faixa etária dos 40 aos 60 anos, com uma média de 44 anos ( Fig. 2 ). A média de idade do sexo masculino foi de 43 e do feminino de 46 anos.

**Fig. 2:** ldade dos participantes.

Em relação ao mecanismo de trauma no grupo dos pacientes que realizaram procedimento cirúrgico com fixador externo circular, observamos uma tendência maior a traumas de alta energia (11 pacientes). Acidentes automobilísticos foram responsáveis por 7 dos 11 casos, com os outros 4 sendo relacionados a quedas de altura. Quanto a esse último subgrupo, os 4 pacientes que apresentaram traumatismos de menor energia, todas as fraturas tiveram relação com quedas do próprio nível. Em função disso observou-se um padrão de 60 de fraturas expostas contra 40% das fechadas no subgrupo que foi submetido a tratamento cirúrgico com fixador externo circular.

Em relação ao mecanismo de trauma no grupo dos pacientes que realizaram procedimento cirúrgico com fixação interna, observamos novamente uma grande tendência a traumas de alta energia (10 pacientes). Acidentes automobilísticos foram responsáveis por 5 dos 10 casos, outros 4 relacionados a quedas de altura e 1 a ferimento por arma de fogo. No subgrupo dos 3 pacientes que apresentaram traumatismos de baixa energia, 2 pacientes apresentaram fraturas relacionadas a agressões físicas e 1 a queda do próprio nível ( Fig. 3 ). Em função disso, observou-se um padrão de aproximadamente 77% de fraturas fechadas contra 23% expostas.

**Fig. 3:** Mecanismo de trauma.

Sobre o padrão de fratura observado, conforme classificação proposta pelo grupo AO, notamos que 60% dos pacientes tratados com fixador externo circular apresentavam fraturas 43C3 com franca cominuição articular, e outros 40% fraturas 43C2. Nenhum dos pacientes tratados pelo método de fixação externa circular apresentaram fraturas do subtipo 43C1.

Usando a mesma classificação, o padrão de fratura observado no grupo de fixação interna, 5 dos pacientes apresentavam fraturas 43C1, 5 fraturas 43C2 e outros 3 fraturas 43C3.

Em ambos os grupos, a lesão associada em 100% dos casos foi a fratura do terço distal da fíbula. Outras lesões visualizadas no grupo de fixação externa circular foram as fraturas do planalto tibial (4 casos), do calcâneo (2 casos), da diáfise da tíbia, do tornozelo contralateral e dos ossos da mão (1 caso em cada). A única outra lesão associada ao grupo de fixação interna documentada foi 1 fratura de clavícula associada.

No que tange a questão de desfecho do grupo dos pacientes tratados com fixador externo circular, observou-se que 5 pacientes apresentaram consolidação plena das fraturas, 1 pseudoartrose e outros 2 consolidações viciosas. As Figs. 4 5 6 7 apresentam exemplos dos grupos tratados com fixação externa circular (grupo I) e com fixação interna (grupo II).

**Fig. 4:** Fratura de pilão tibial (AO 43C2) no pré-operatório de fixação com placa e parafuso.

**Fig. 5:** Fratura de pilão tibial (AO 43C2) no pós-operatório de fixação com placa e parafuso.

**Fig. 6:** Fratura de pilão tibial (AO 43C2) no pré-operatório de fixação externa circular.

**Fig. 7:** Fratura de pilão tibial (AO 43C2) no pós-operatório de fixação externa circular.

Dos pacientes relatados no estudo em questão, 6 ainda se encontram em acompanhamento, mas evoluem de maneira satisfatória, com sinais radiográficos de consolidação. Um único paciente perdeu seu seguimento ( Fig. 8 ).

**Fig. 8:** Desfecho fixador externo circular.

No que tange a questão de desfecho dos casos em que foi realizado redução aberta e fixação interna observou-se que 10 pacientes apresentaram consolidação plena das fraturas, 1 dos casos evoluiu com pseudoartrose e também 1 caso apresentou consolidação viciosa. Por fim, um dos casos apresentou infecção franca na topografia necessitando retirada do material de síntese e múltiplas intervenções cirúrgicas ( Fig. 9 ).

**Fig. 9:** Desfecho placa e parafuso.

A fim de tornar mais palpável a avaliação funcional dos pacientes conforme a escala da American Orthopedic Foot & Ankle Society (AOFAS) para tornozelo e retropé ( Fig. 10 ), subdividimos o grupo avaliado da seguinte forma:

**Fig. 10:** Escala da American Orthopedic Foot & Ankle Society (AOFAS) para tornozelo e retropé (total de 100 pontos).

- Grupo 1: de 0 a 20 pontos
- Grupo 2: 20 a 40 pontos
- Grupo 3: 40 a 60 pontos
- Grupo 4: 60 a 80 pontos
- Grupo 5: 80 a 100 pontos

Dessa forma, observamos que a distribuição dos grupos foi a seguinte: grupo 1–2 pacientes; grupo 2–6 pacientes; grupo 3–7 pacientes; grupo 4–8 pacientes (este grupo apresentou a maior prevalência); e grupo 5–5 pacientes.

Em relação ao subgrupo do tratamento com fixador externo Ilizarov, que continha 15 pacientes, observou-se a seguinte distribuição: grupo 1–1 paciente; grupo 2–3 pacientes; grupo 3–4 pacientes; grupo 4–4 pacientes; e grupo 5–3 pacientes.

Em relação ao subgrupo do tratamento com placa e parafusos, que continha 13 pacientes, observou-se a seguinte distribuição: grupo 1 – 1 paciente; grupo 2–3 pacientes; grupo 3–3 pacientes; grupo 4–4 pacientes; e grupo 5–2 pacientes.

## Discussão

O presente estudo encontrou uma prevalência do sexo masculino, corroborando com a literatura. Entretanto, a prevalência da faixa etária esteve acima da média da literatura descrita por Marsh e Saltzaman. 5 No estudo de Pimenta et al., 8 observou-se uma média de idade de 42 anos, similar a deste trabalho.

No grupo dos pacientes tratados com fixador externo circular, a taxa de fraturas expostas ficou acima dos percentis exibidos na literatura. 9 Em contrapartida, o grupo tratado com fixação interna apresentou taxa de lesão de partes moles com exposição da fratura bem abaixo da bibliografia, na qual a taxa de fraturas expostas está em cerca de 50% dos casos.

Em nosso serviço, o principal mecanismo de trauma foi o acidente automobilístico, seguido por queda de altura, o que corresponde ao encontrado literatura. Além disso, foi possível observar que a maior taxa de exposição de fratura entre os 28 pacientes analisados ocorreu no grupo classificado como AO 43C2, representando 60% dos pacientes, comparada aos 38% do grupo AO 43C3. Essas taxas diferem da literatura, a qual encontra um maior percentil de fratura exposta no grupo com a fratura mais complexa, classificada como AO 43C3. 2

Ao avaliar se houve presença ou não da fratura da fíbula e correlacionar esse dado entre os grupos de alta e baixa energia, não encontramos diferença estatisticamente significante. A ausência de evidência deu-se uma vez que, em nosso estudo, a totalidade dos pacientes apresentou fratura da fíbula distal associada, mesmo naqueles com menor energia do trauma. Além disso, a literatura define que as fraturas de tálus e calcâneo ipsilaterais associadas a fratura de pilão tibial são extremamente raras. Contudo, encontramos 2 pacientes que apresentaram fratura de calcâneo associada, podendo influenciar negativamente os resultados a longo prazo. 9 10

No que tange os desfechos radiográficos, verifica-se que os estudos avaliam os resultados de formas variadas. Em nosso estudo, concluímos que os pacientes tratados por fixador externo circular que já apresentam desfecho radiográfico definido tiveram uma taxa de 62,5% de consolidação da fratura, 25 de consolidação viciosa e 12,5 pseudoartrose. No grupo tratado com placa e parafuso que foram possíveis de serem avaliados, 84% apresentaram consolidação, 8% consolidação viciosa, 8% pseudoartrose. Não foi possível a avaliação total nos demais, levando à exclusão da pesquisa.

Em relação à média geral de nossa série, considerando todos os 28 pacientes eleitos, obtivemos uma média de 55,7 pontos na escala AFAOS (grupo fixador externo circular: 56,6; grupo fixação interna: 54,6). Esse valor é inferior à média de 65 pontos obtidos na série avaliada por Moura Junior et al. 2

## Conclusão

Este estudo avaliou retrospectivamente 28 casos de fratura do pilão tibial, em que 15 casos foram submetidos a tratamento cirúrgico com fixador externo circular e outros 13 foram submetidos a tratamento cirúrgico com fixação interna por placa (do tipo trevo, simples, sem mecanismo de bloqueio) e parafusos. Constatamos uma ampla gama de variantes que influenciam diretamente nas características de cada fratura e também do seu desfecho clínico-radiográfico.

Apesar de existirem muitas possibilidades terapêuticas para fraturas do pilão tibial, os métodos de fixação interna por placa (não bloqueadas) e parafusos, assim como o método de fixação externa circular, ainda são os mais disponíveis, principalmente em hospitais do sistema único de saúde. Portanto, é muito importante e inevitável a discussão destes métodos.

É imprescindível ressaltar que o perfil das fraturas e dos pacientes no grupo fixador externo circular e no de fixação interna por placa e parafusos tende a ser bastante heterogêneo. Isso ocorre uma vez que fraturas de pior classificação (conforme classificação AO) e mais frequentemente associadas a lesões de partes moles (sejam elas a exposição da fratura ou até mesmo edema importante ou flictenas) costumam ser manejadas com fixação externa circular. Enquanto isso, pacientes que apresentam fraturas de classificação de menor gravidade e têm menor incidência de lesão de partes moles tendem a ser manejados por redução aberta e fixação interna. Os desfechos clínicos e radiográficos tendem a ser similares entre os grupos, apesar das particularidades de cada método.

Conclui-se a extrema importância da realização de uma avaliação atenta, individualizada e detalhada. Isso possibilita uma escolha terapêutica mais assertiva, que resultará no melhor desfecho funcional para as particularidades de cada caso, assim como menores taxas de impacto socioeconômico para portadored de fraturas do pilão tibial.
